# Aortic Arch Incision and Closure Technique (AICT) for Proximal Fixation of the Frozen Elephant Trunk

**DOI:** 10.3390/jcm15051861

**Published:** 2026-02-28

**Authors:** Shun-Ichiro Sakamoto, Kenji Suzuki, Yoshiyuki Watanabe, Motohiro Maeda, Tomohiro Murata, Atsushi Hiromoto, Yosuke Ishii

**Affiliations:** 1 Department of Cardiovascular Surgery, Nippon Medical School Musashi Kosugi Hospital, Kawasaki 211-8533, Japan; suzuki@nms.ac.jp (K.S.); yoshi-w@nms.ac.jp (Y.W.); m-maeda@nms.ac.jp (M.M.); t-murata@nms.ac.jp (T.M.); a-hiromoto@nms.ac.jp (A.H.); 2 Department of Cardiovascular Surgery, Nippon Medical School, Tokyo 113-8602, Japan; yosuke-i@nms.ac.jp

**Keywords:** frozen elephant trunk, aortic arch, thoracic aortic aneurysm, surgical technique, endoleak

## Abstract

**Background**: To describe an aortic arch incision and closure technique (AICT) for proximal fixation of a frozen elephant trunk (FET) and to report early outcomes. **Methods**: We retrospectively reviewed 15 consecutive patients who underwent distal arch repair with an FET using AICT (mean age 77 ± 7 years; 14 men). Indications were distal arch aneurysm (*n* = 12), acute Stanford type B dissection (*n* = 2), and distal arch enlargement after thoracic endovascular aortic repair (*n* = 1). Under circulatory arrest, an oblique arch aortotomy was created, the FET was deployed antegrade, trimmed, and sutured to the native aortic wall during simultaneous closure, allowing extended posterior fixation. Clinical outcomes and postoperative computed tomography were assessed. **Results**: No ischemic complications related to graft kinking or thrombosis, reoperation for bleeding, stroke, spinal cord ischemia, or organ failure occurred. One patient died of pneumonia on postoperative day 47 (6.7%). Cervical branch reconstruction was required in 12 patients (80%), whereas two patients with type III arch morphology and acute angulation were treated without debranching via a Zone 3 aortotomy. At a median follow-up of 29 months, no proximal endoleak was observed; one distal endoleak occurred without reintervention. Coronary bypass grafts remained patent in all patients with concomitant or prior CABG. **Conclusions**: AICT provided secure proximal FET fixation and arch closure while preserving the ascending aorta, offering an alternative to total arch replacement in selected distal arch pathologies.

## 1. Introduction

Aortic stenting for distal arch aortic lesions has become increasingly popular in recent years. The advantages of arch replacement using frozen elephant trunks (FET) include elimination of the need for peripheral anastomosis, the ability to perform suturing proximally away from the recurrent laryngeal nerve, and prevention of endoleak by anastomosing a three-branch prosthetic graft [1,2,3]. On the other hand, graft replacement that includes the cervical branches is required even when the ascending aorta is in good condition. Although debranching TEVAR is less invasive, anatomical factors such as arch morphology and branch positions determine the proximal landing zone. In such settings, stent placement can be challenging in cases of ascending aortic calcification, the presence of functional grafts after coronary artery bypass surgery, or a gothic arch, and the risk of type IA endoleak may increase [4,5,6].

Several incision-based open stent grafting approaches have been reported previously [7,8]. However, we developed a modified technique, termed the arch incision and closure technique (AICT), in which an oblique aortic arch aortotomy is created to allow proximal fixation of the frozen elephant trunk during simultaneous arch closure. A key feature of AICT is that the posterior fixation line can be extended proximally toward the region just distal to the left common carotid artery (Zone 1 region), whereas the anterior fixation corresponds to the aortotomy closure line.

Unlike conventional strategies, AICT does not require ascending aortic replacement and may address limitations associated with existing stenting procedures. The purpose of the present study was to evaluate the clinical outcomes of AICT across a range of distal arch pathologies and aortic arch morphologies.

## 2. Materials and Methods

### 2.1. Patients

Since December 2021, a total of 15 patients (mean age, 77 ± 6.8 years; 14 males) underwent AICT (Table 1). AICT was indicated for patients with any of the following conditions: a proximal landing zone of 0 for TEVAR, marked aortic arch angulation, significant calcification or atheroma, a history of coronary artery bypass grafting or planned concomitant bypass surgery, type I endoleak after TEVAR, or small-diameter access vessels. Contraindications for AICT included a severely fragile or infected aortic arch wall unsuitable for suturing, as well as a marked diameter mismatch between the proximal and distal aorta.

The underlying pathologies included distal aortic arch aneurysms (*n* = 12), Stanford type B acute aortic dissection (*n* = 2), and aortic enlargement after thoracic endovascular aortic repair (*n* = 1). One patient had undergone prior coronary artery bypass grafting (CABG). The surgical procedure consisted of AICT alone in 13 cases, while CABG and left atrial appendage closure were performed as concomitant procedures in one case each.

### 2.2. Surgical Procedure

**Figure 1:**
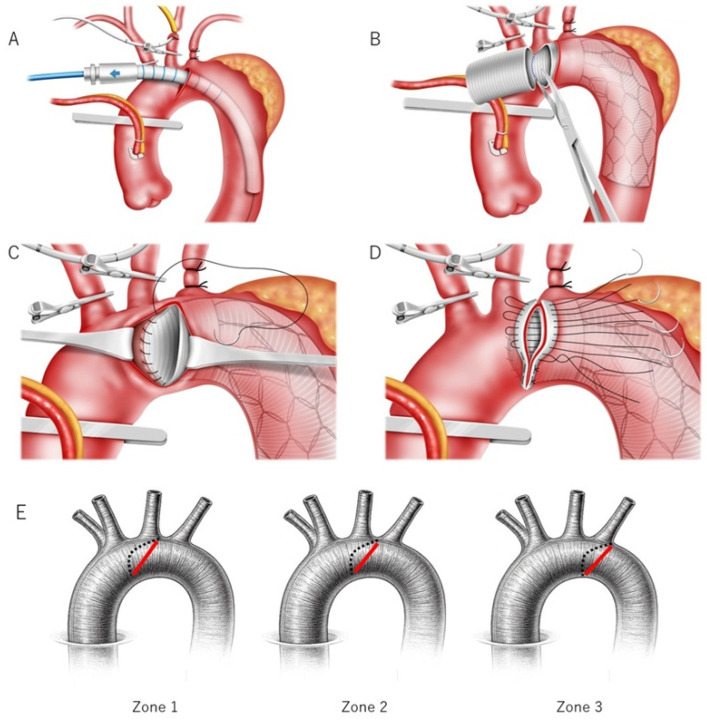
Arch incision and closure technique (AICT). (**A**) Oblique aortotomy of the aortic arch performed under circulatory arrest. (**B**) After deployment of the frozen elephant trunk, the proximal non-stented graft portion is trimmed. (**C**) Posterior graft fixation is performed first; importantly, the posterior suture line can be extended more proximally than the anterior aortotomy closure line, toward the region just distal to the left common carotid artery (Zone 1 region), thereby increasing the effective proximal anchoring length. (**D**) The anterior portion of the graft is secured, and the aortotomy is closed with felt reinforcement. (**E**) Schematic illustration of graft fixation lines and incision patterns. The red line indicates the anterior fixation line, which corresponds to the aortic incision and closure line, whereas the dotted line indicates the extended posterior fixation line. Depending on the arch anatomy and the extent and characteristics of the aortic pathology, the oblique incision is initiated distal to the left subclavian artery (Zone 3), proximal to the left subclavian artery (Zone 2), or distal to the left common carotid artery (Zone 1).

In 14 cases, a median sternotomy was used, and in one case, right-sided thoracotomy with a clamshell extension was employed due to mediastinal deviation to the right (Case 13). Cardiopulmonary bypass was initially established using ascending aortic cannulation in combination with common femoral artery and bicaval cannulation in the early phase of AICT. In more recent cases, ascending aortic cannulation was avoided to maintain an adequate operative field, and arterial inflow was achieved via right axillary artery cannulation using an 8-mm polytetrafluoroethylene (PTFE) graft (*n* = 7, 46%), together with common femoral artery and bicaval cannulation. An additional 8-mm PTFE graft was sutured to the left axillary artery when intraoperative debranching was anticipated. The ascending aorta was clamped using a flexible aortic clamp, and cardioplegia was given into the heart through the aortic root and coronary sinus. Under moderate hypothermia (26 °C) and circulatory arrest, an oblique incision was made in the aortic arch. Selective cerebral perfusion was used in all cases, with direct cannulation of the left common carotid artery. During the deployment of the FET, retrograde blood flow was temporarily provided from the femoral artery to evacuate air between the stent and the aortic wall [10]. The graft was trimmed at the aortic incision site, sutured to the posterior wall, and fixed to the anterior wall along with the aortic closure (Figure 1).

### 2.3. Postoperative Imaging Follow-Up

Contrast-enhanced CT was performed prior to discharge, at 1 month, 6 months, and at 1 year postoperatively. Thereafter, CT was performed annually to check for the presence of pseudoaneurysms or endoleaks.

### 2.4. Aortic Arch Morphometric Assessment

**Figure 2:**
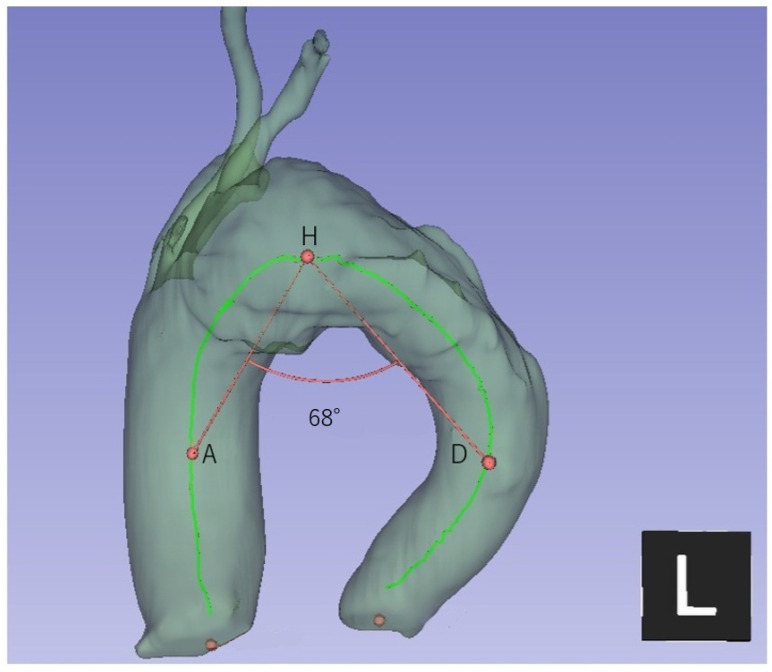
Measurement of aortic arch angle (Case 6). Using 3D Slicer, the aortic centerline is extracted and the angle is measured between the ascending and descending segments on the sagittal view. The colored lines indicate the centerlines of the ascending and descending aorta, and the angle is formed at the highest point of the aortic arch.

Native aortic arch morphometry was assessed using computed tomography (CT) and 3D multiplanar reconstruction. The aortic arch angle was measured on the sagittal plane as the angle formed by the centerlines of the ascending and descending aorta and the highest point of the aortic arch, as previously described [11]. The angle was defined using three anatomical points: two points placed on the centerlines of the ascending and descending aorta at the level of the pulmonary artery bifurcation, and one point placed at the highest point of the aortic arch in the sagittal plane (Figure 2). In addition, aortic arch morphology was classified according to the conventional Type I–III arch configuration based on the vertical distance from the origin of the brachiocephalic trunk to the top of the aortic arch, as described by Demertzis et al. [9]. All measurements were performed using 3D Slicer (version 5.8.1).

### 2.5. Statistical Analysis

Continuous variables are presented as mean ± standard deviation or median (interquartile range), as appropriate. Categorical variables are presented as counts and percentages. Given the descriptive nature and sample size, no hypothesis testing was performed.

## 3. Results

**Figure 3:**
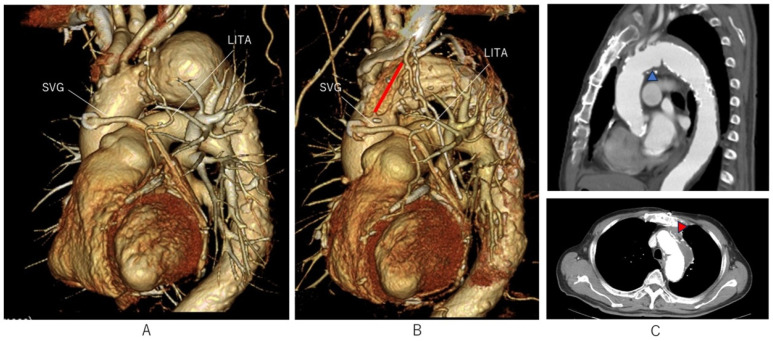
Example of a distal arch aneurysm with patent bypass grafts. (**A**) Preoperative three-dimensional CT reconstruction demonstrating patent LITA and SVG grafts. (**B**) Postoperative reconstruction showing preservation of the ascending aorta and proximal fixation at the aortotomy closure site (red line). (**C**) Postoperative CT images confirming secure incorporation of the proximal graft within the native arch. The sagittal view (**upper panel**) demonstrates the proximal fixation site (blue arrowhead), and the axial view (**lower panel**) shows the sutured closure line reinforced with felt (red arrowhead).

Baseline aortic morphology, operative details, intraoperative findings, and surgical outcomes are summarized in Table 1 and Table 2. Cervical debranching was required in 12 cases (80%), and two debranching procedures were performed in two cases. Type III arch morphology was present in 9 patients (60%). Cervical branch reconstruction was not required in two cases (13%), both of which were achieved with a Zone 3 aortotomy. These patients exhibited a type III arch morphology with acute aortic arch angles (<60°). No postoperative complications, including ischemic events attributable to graft kinking or intraluminal thrombosis, reoperation for bleeding, pseudoaneurysm at the aortic suture line, cerebral or spinal ischemia, or organ failure, were observed. All patients were discharged alive except for one who died of pneumonia on the 47th postoperative day (mortality rate 6.7%). During the follow-up period (median 29 months), there were no cases of type IA endoleak. Only one case of type IB endoleak was observed, but no reintervention was necessary. All grafts used for concomitant or prior CABG were patent postoperatively (Figure 3).

## 4. Discussion

The Elephant Trunk technique, first described by Borst et al. in 1983, was designed to facilitate staged repair of extensive thoracic aortic aneurysms by placing a free graft segment into the descending aorta [12]. In the 1990s, Kato et al. introduced the concept of open stent grafting, integrating a stented graft into the distal aortic arch to extend the repair into the descending aorta [1]. This concept was later refined and popularized in Europe by Karck and colleagues, who termed the procedure the FET technique [13].

AICT is a simplified FET procedure that omits replacement of the ascending aorta and reconstruction of the supra-aortic branches. The origin of this approach lies in the less invasive quick stenting (LIQS) technique reported by Hata et al., which was designed for the treatment of distal arch aneurysms [7]. In all cases, the proximal end of the stent was sutured and fixed distal to the left subclavian artery, without branch reconstruction, under mild hypothermic circulatory arrest and without selective cerebral perfusion. In contrast, the AICT adjusts the site of aortic incision according to the individual anatomy of each patient, lowers the circulatory arrest temperature, and employs selective cerebral perfusion. Because the graft is anastomosed at the incision site, this method is not only technically straightforward but also provides superior safety in terms of cerebral protection. Hioki et al. have reported a similar approach in a case report [8]; however, to date, there have been no reports describing the application of this method to a series of patients with various aortic pathologies.

Recent advances in branched endografts and cerebral protection strategies have improved the outcomes of Zone 0 TEVAR [14]. Nevertheless, endovascular repair in the proximal arch remains challenging in selected patients, particularly those with severe arch angulation, heavy calcification or shaggy aorta, and functional coronary bypass grafts, where the risks of embolic stroke or type IA endoleak may still be substantial. In such situations, AICT may provide an alternative strategy by enabling direct proximal fixation of the frozen elephant trunk under open surgical control while preserving the ascending aorta.

**Figure 4:**
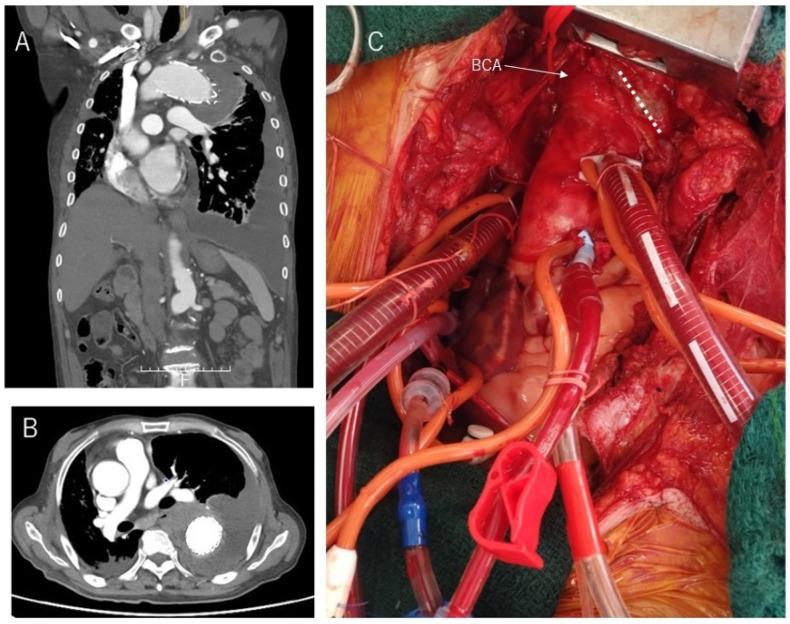
AICT performed via right thoracotomy in a patient with aneurysmal enlargement after TEVAR. (**A**) Preoperative sagittal computed tomography demonstrating aneurysmal expansion of the distal arch secondary to a type I endoleak after previous TEVAR, with a proximal stent-graft “beak sign” indicating incomplete apposition in the sharply angulated arch. (**B**) Axial CT image showing the enlarged distal arch aneurysm and the malapposed proximal stent graft. (**C**) Intraoperative view through a right thoracotomy approach. The dotted line indicates the aortotomy closure (suture) line, which was created adjacent to the brachiocephalic artery (BCA).

AICT is not designed to reduce circulatory arrest time, but to limit the extent of arch reconstruction in selected patients. Furthermore, it may reduce procedural cost by avoiding the routine use of a four-branched graft. Although FET devices equipped with four branches have recently become available and offer improved convenience, they may not outweigh the benefits of AICT in cases where the ascending aorta has a favorable morphology [15,16]. This is because the extent of aortic resection must be increased when using such devices, resulting in greater surgical invasiveness for the patient. Furthermore, the localized aortic incision and direct suturing performed in AICT is useful in a variety of clinical situations. In patients who have undergone prior CABG, AICT enables preservation of functional grafts (Figure 3). The rationale is not related to technical difficulty of proximal anastomosis, but rather to minimizing the risk of graft injury and embolic complications in redo or complex settings. It also allows secure inflow for grafts when CABG is performed concomitantly, by preserving both the carotid branches and the ascending aorta. The AICT is advantageous not only through a median sternotomy but also in restricted operative fields such as a right thoracotomy. In Case 13, the mediastinum was markedly displaced to the right due to aneurysmal enlargement secondary to an endoleak after TEVAR, allowing AICT to be performed safely via a right thoracotomy (Figure 4).

We also performed AICT in patients with aortic dissection; all of these cases involved Stanford type B dissection, and no dissected layers were present at the anastomotic sites. Liu et al. reported favorable outcomes in Stanford type A aortic dissection by preserving the supra-aortic branches through trimming the proximal portion of the FET to exclude the branch orifices, and then securing this segment to the aortic wall using an inclusion technique [17]. In our series, only three patients (20%) underwent AICT without debranching; however, their findings indicate that non-debranching AICT may be feasible in a broader range of cases. In contrast, the inclusion technique—suturing the prosthetic graft from the aortic intimal surface to the aortic wall—is required in certain situations, particularly in surgical strategies that make active use of this method. The inclusion suture technique used in AICT was first described by Bentall and De Bono in 1968; however, it has been associated with the development of postoperative pseudoaneurysms [18]. Because the native aorta is not transected and the prosthetic graft is anastomosed only to the intimal layer, blood can accumulate in the potential space between the graft and the aortic wall, creating tension on the anastomosis and predisposing it to dehiscence [19,20].

To prevent such suture failure, full-thickness anastomosis between the graft and the native aortic wall is considered essential. In the present series, the posterior portion of the non-stented segment of the prosthesis was deliberately trimmed longer than the aortotomy so that it could be positioned above a flat handheld retractor placed posterior to the aorta during posterior-wall suturing. This technique served two purposes: (1) to avoid injury to the trachea, and (2) to ensure reliable full-thickness graft fixation by allowing the needle to be advanced until it contacted the retractor on the adventitial side of the aorta. The anterior and lateral aspects of the graft were then trimmed to match the aortotomy line and secured during closure of the aorta. Using this approach, no postoperative pseudoaneurysms occurred in any patient. Postoperative computed tomography demonstrated complete apposition of the proximal graft to the native arch wall at the closure site, without evidence of proximal endoleak or anastomotic dilatation.

The use of a FET in cases with sharp (acute) angulation of the aortic arch has been reported to cause complications such as rupture, pseudoaneurysm, and endoleak. In particular, an aortic isthmus that ascends superiorly is considered to carry a higher risk of such complications [21]. In the present cohort, the aortic arch angle was 65.4 ± 6.2 degrees. Since the mean angle in healthy individuals has been reported to be approximately 74 degrees, our cases exhibited more acute angulation than normal. Although type III morphology—characterized by a superiorly directed aortic isthmus and known to be associated with higher FET-related complication rates—accounted for 60% of our patients, only one case of late type I endoleak occurred postoperatively. Thus, the aortic morphology did not constitute a significant risk factor.

**Figure 5:**
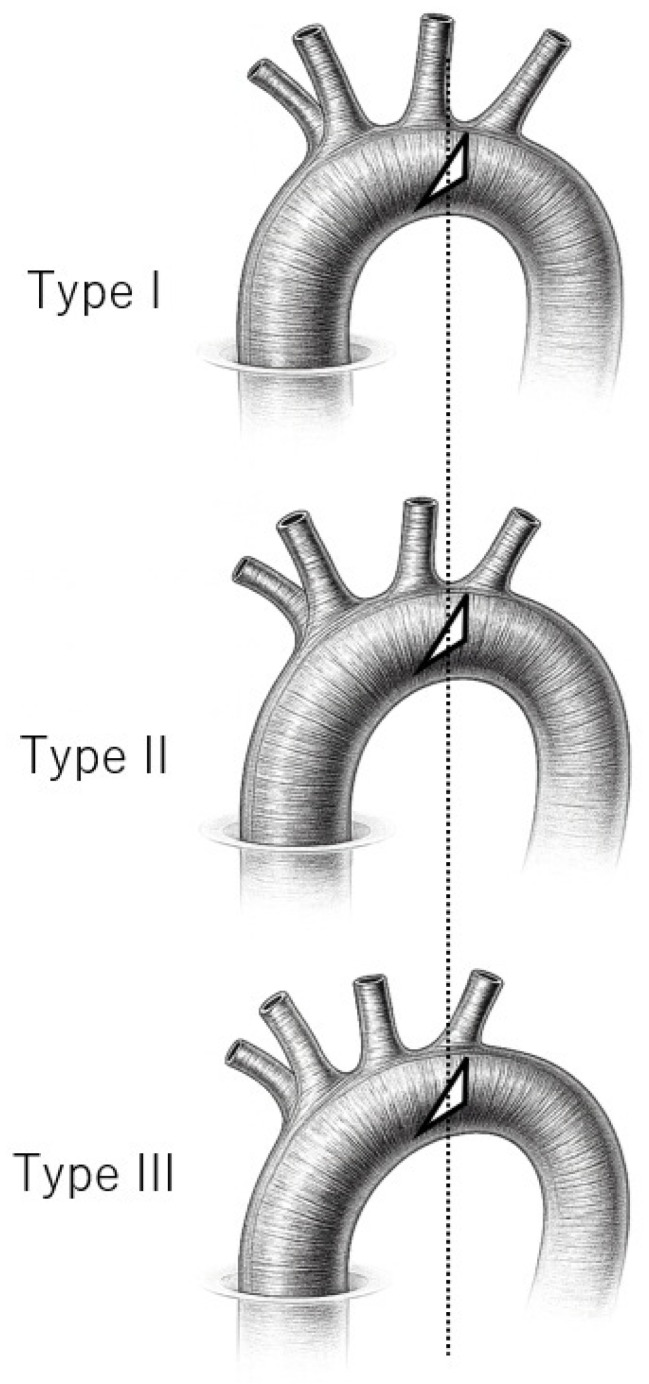
Aortic arch morphology and aortotomy position in AICT. Schematic illustration of the Type I–III arch configuration and the corresponding location of the oblique aortotomy. The triangular area indicates the anterior aortotomy closure line. The incision level shifts relative to the supra-aortic branches according to arch type.

Rather, in type III anatomy, it is often easier to create a Zone 3 aortotomy within the same operative field, making AICT feasible without the need for cervical debranching. Notably, all cases in which debranching was unnecessary in this study had a type III aortic arch with acute arch angulation. These findings may suggest that, in selected patients with type III morphology and acute aortic arch angulation, AICT with a Zone 3 aortotomy can potentially be performed without supra-aortic branch reconstruction. In contrast, even when a Zone 3 aortotomy was feasible, patients with type I anatomy still required at least one debranching procedure. This may reflect the increased distance and limited exposure of the posterior arch in type I configuration, which can make posterior-wall suturing to exclude the left subclavian artery more technically challenging (Figure 5).

The selection and deployment strategy of a FET are critical factors influencing postoperative complications. Frozenix^®^ (Japan Lifeline Co., Ltd., Tokyo, Japan) was used as the Frozen Elephant Trunk (FET) prosthesis in all cases. When using the Frozenix device, we selected the graft size according to the manufacturer’s recommended oversizing, corresponding to 105–120% of the native aortic diameter. The distal landing zone was planned at a site with a diameter comparable to that of the proximal aorta, ensuring a sealing length of 2–3 cm from the aneurysmal segment [22]. Although the optimal strategy may vary depending on the underlying aortic pathology, this approach is generally consistent with previously reported techniques using FET devices other than Frozenix, and there is growing consensus regarding standardized deployment strategies to prevent endoleak, distal stent graft-induced new entry, and spinal cord ischemia [23,24,25]. In the present series, endoleak occurred in only one case. Kandola et al. reported that a sealing length of less than 3 cm and graft oversizing of 110% or less constitute undersizing, both of which are associated with an increased risk of endoleak due to inadequate sealing [26]. In our endoleak case, the patient had a shaggy aorta with mural thrombus. Although a deeper insertion of the FET than usual was attempted to achieve landing in a relatively normal segment, the graft size selected was at the lower limit of the recommended range. This factor may have contributed to the development of endoleak.

The principal advantage of the Frozen Elephant Trunk (FET) technique is the absence of type IA endoleak. However, graft kinking—particularly stenosis of the non-stented segment—has been reported and may lead to lower-limb ischemia with intermittent claudication, graft thrombosis, multi-organ septic emboli, and multi-organ ischemic injury. Based on a comprehensive review of the existing literature, a non-stented graft length of approximately 7–8 cm has been suggested to minimize the risk of graft kinking [27]. In contrast, for the Frozenix, even in multicenter studies, only one case (1.6%) of early postoperative kinking has been reported. Nevertheless, to ensure optimal conformity of the stented segment to the native aorta, it is generally recommended that the non-stented portion be kept as short as possible. In the present series, the non-stented segment was limited to 3–4 cm, and no cases of graft kinking were observed. Shortening the non-stented segment reduces graft redundancy, facilitates trimming, and allows easier sutured fixation [28]. Accordingly, the stent length was determined based on the planned insertion length and the length of the non-stented segment. As a result, a 9-cm stent was used in 80% of the cases in this series.

## 5. Conclusions

AICT enables secure proximal fixation of the frozen elephant trunk while reliably preserving the ascending aorta. It may serve as a useful, limited open alternative to total arch replacement in selected distal arch pathologies, particularly when ascending aortic manipulation should be avoided.

## Figures and Tables

**Table 1:** Patient characteristics.

Case	Sex	Age (y)	Aortic Disease	Operation Urgency	Previous Operation	Aortic Arch Type	Aortic Arch Angle (°)
1	M	83	AAD	emergent	None	II	75
2	M	86	TAA	elective	None	III	70
3	M	73	AAD	emergent	None	II	64
4	M	81	TAA	elective	None	III	61
5	M	83	TAA	elective	None	III	67
6	F	74	TAA	elective	None	III	68
7	M	60	TAA	elective	None	I	73
8	M	76	TAA	elective	CABG	III	59
9	M	84	TAA	elective	None	II	61
10	M	82	TAA	elective	None	III	64
11	M	77	TAA	elective	None	III	58
12	M	77	TAA	elective	None	I	76
13	M	65	TAA	elective	TEVAR	III	57
14	M	77	TAA	elective	None	II	72
15	M	75	TAA	elective	None	III	62

Abbreviations: AAD, acute aortic dissection; TAA, thoracic aortic aneurysm; CABG, coronary artery bypass grafting; TEVAR, thoracic endovascular aortic repair. Aortic arch type (I–III) was classified according to the conventional arch configuration described by Demertzis et al. [9]. Aortic arch angle is expressed in degrees (°).

**Table 2:** Intraoperative variables and postoperative events.

Case	Combined Procedures	FET Configuration	Debranched Vessels (*n*)	Aortotomy Closure Zone	CA Time (min)	Operation Time (min)	Complications/Mortality
1	None	37-120-160	1	Zone 2	36	285	None
2	None	33-90-130	2	Zone 1	46	301	None
3	None	27-90-120	1	Zone 2	69	342	hospital death (POD 47)
4	None	31-120-150	1	Zone 2	50	404	type IB endoleak (POM 12)
5	None	35-90-120	1	Zone 2	33	390	None
6	None	31-90-120	1	Zone 2	41	433	None
7	CABG	29-90-120	1	Zone 3	42	431	None
8	None	31-90-120	0	Zone 3	62	485	None
9	None	29-90-120	1	Zone 2	35	344	None
10	None	31-90-130	1	Zone 2	37	411	None
11	LAAC	31-90-120	0	Zone 3	55	316	None
12	None	31-90-130	1	Zone 3	42	395	None
13	None	39-90-120	1	Zone 2	56	329	None
14	None	35-120-150	2	Zone 1	55	395	None
15	None	31-90-120	1	Zone 2	53	354	None

Abbreviations: CA, circulatory arrest; CABG, coronary artery bypass grafting; LAAC, left atrial appendage closure; FET, frozen elephant trunk; POD, postoperative day; POM, postoperative month. FET configuration is presented as diameter, stent, and insertion lengths (mm), in this order.

## Data Availability

The data presented in this study are available upon request from the corresponding author. The data are not publicly available due to privacy/ethical restrictions.

## Abbreviations

AICT	arch incision and closure technique
FET	frozen elephant trunk
TEVAR	thoracic endovascular aortic repair
CABG	coronary artery bypass grafting
LAAC	left atrial appendage closure
TAA	thoracic aortic aneurysm
AAD	acute aortic dissection
CA	circulatory arrest
CT	computed tomography
PTFE	polytetrafluoroethylene
BCA	brachiocephalic artery
LITA	left internal thoracic artery
SVG	saphenous vein graft
POD	postoperative day
POM	postoperative month
